# Impaired Glucose Tolerance is Associated with Enhanced Platelet-Monocyte Aggregation in Short-Term High-Fat Diet-Fed Mice

**DOI:** 10.3390/nu11112695

**Published:** 2019-11-07

**Authors:** Zibusiso Mkandla, Tinashe Mutize, Phiwayinkosi V. Dludla, Bongani B. Nkambule

**Affiliations:** 1 School of Laboratory Medicine and Medical Sciences (SLMMS), College of Health Sciences, University of KwaZulu-Natal, Durban 4000, South Africa; 217063126@stu.ukzn.ac.za (Z.M.); 217063119@stu.ukzn.ac.za (T.M.); nkambuleb@ukzn.ac.za (B.B.N.); 2 Biomedical Research and Innovation Platform (BRIP), South African Medical Research Council, Tygerberg 7505, South Africa; 3 Department of Life and Environmental Sciences, Polytechnic University of Marche, 60131 Ancona, Italy

**Keywords:** high fat diet, metabolic dysregulation, platelets, monocytes, hypercoagulation, inflammation

## Abstract

High-fat diet (HFD) feeding is known to induce metabolic dysregulation, however, less is known on its impact in promoting the hypercoagulable state. This current study aimed to evaluate platelet-monocyte aggregate (PMA) formation following short-term HFD feeding. This is particularly important for understanding the link between inflammation and the hypercoagulable state during the early onset of metabolic dysregulation. To explore such a hypothesis, mice were fed a HFD for 8 weeks, with body weights as well as insulin and blood glucose levels monitored on a weekly basis during this period. Basal hematological measurements were determined and the levels of spontaneous peripheral blood PMAs were assessed using whole blood flow cytometry. The results showed that although there were no significant differences in body weights, mice on HFD displayed impaired glucose tolerance and markedly raised insulin levels. These metabolic abnormalities were accompanied by elevated baseline PMA levels as an indication of hypercoagulation. Importantly, it was evident that baseline levels of monocytes, measured using the CD14 monocyte marker, were significantly decreased in HFD-fed mice when compared to controls. In summary, the current evidence shows that in addition to causing glucose intolerance, such as that identified in a prediabetic state, HFD-feeding can promote undesirable hypercoagulation, the major consequence implicated in the development of cardiovascular complications.

## 1. Introduction

Chronic platelet activation has been associated with a sustained pro-inflammatory response and an increased risk of cardiovascular complications [1]. Monocytes, via the surface membrane P-selectin glycoprotein ligand-1 (PSGL-1), bind to P-selectin expressed on the activated endothelial surface-mediated interaction [2]. This represents the early events in the pathophysiological mechanisms leading to atherosclerosis under dysregulated metabolic complications such as type 2 diabetes mellitus (T2DM) [3]. In a similar manner, activated platelets are able to bind circulating peripheral blood leucocytes via P-selectin and PSGL-1 interactions, forming platelet-leukocyte aggregates (PLAs) [4,5]. The binding of P-selectin to its counter-receptor PGSL1 induces leukocyte tethering and firm adhesion of monocytes to the endothelium [6,7]. The endothelium serves an important role in the hemostatic system, and it appears to be greatly impacted by lifestyle modifications such as high-fat diet feeding (HFD) that are associated with enhanced metabolic stress, and this may lead to the development of prothrombotic events [7].

It is well established that overfeeding, especially excess intake of saturated fatty acids coupled with physical inactivity, are factors which contribute to the development of metabolic syndrome [1]. Recently, we reviewed evidence showing that inflammation together with other consequences such as oxidative stress are the foremost causal factors implicated in the aggravation of metabolic disease-associated complications [8]. In conditions of metabolic dysregulation, elevated expression of pro-inflammatory markers such as monocyte chemoattractant protein-1 (MCP-1), tumor necrosis factor-α (TNF-α), interleukin-8 (IL-8), IL-1β, and cyclooxygenase-2 (COX-2) is concomitant with the formation of platelet monocyte aggregates (PMA) [8]. In fact, previous studies have shown that platelets preferentially bind to monocytes, thus PMAs are regarded as stable markers of platelet activation [3,5,9]. These interactions provide a link between the inflammatory and thrombotic responses involved in conditions such as T2DM where elevated levels of PMAs have been reported [10,11,12]. The aim of this study was to assess the impact of HFD-feeding on PMA formation in mice, to better understand the link between pro-inflammatory status and hypercoagulable state under dysregulated metabolic condition. Furthermore, investigating the impact of short-term HFD feeding could help identify essential pathophysiological mechanisms, implicating inflammation during the onset of metabolic complications such as T2DM. Also, of interest is the influence of short-term HFD feeding on the dysregulation of haematological indices, since this consequence has been involved in the aggravation of metabolic disease-associated complications [13]. This is especially important since progression conditions like T2DM can contribute to the malabsorption of iron by the intestines, causing reduced haemoglobin levels [13].

## 2. Materials and Methods

### 2.1. Study Design

Five-week-old C57BL/6 male mice were purchased from the Jackson’s Laboratories (Sacramento, USA) and housed, individually in a cage, at the University of KwaZulu-Natal (UKZN) biomedical research unit (BRU). The C57BL/6 mice strain is well characterised and has been shown to become glucose intolerant when kept on a HFD [13]. Animals were handled according to the principles of Laboratory Animal Care of the National Society of Medical Research and the National Institutes of Animal Care and Use of Laboratory Animals of the National Academy of Sciences (National Institute of Health publication 80–23, revised 1978). Ethical clearance was granted by the UKZN animal research ethics committee (AREC), ethics registration number AREC/086/016.

Briefly, 16 mice were randomly allocated to receive two experimental diets. The control group (*n* = 8) was fed with the low-fat diet containing 10 Kcal% derived from fat (Research Diets, New Brunswick, NJ, USA). The study group (*n* = 8) was fed with the HFD containing 60% Kcal% derived from fat (Research Diets, New Brunswick, NJ, USA). An overview of diet composition for the control (low-fat diet) and HFD groups is displayed in Table 1. During the study, mice, both controls and HFD fed mice, were monitored for body weights, as well as blood glucose and insulin levels for 8 weeks. Furthermore, the oral glucose tolerance test was performed, and all glucose measurements were performed using the OneTouch^®^Select^®^ handheld glucometer (LifeScan Inc., Milpitas, CA, USA).

### 2.2. Blood Collection for Haematology Characteristics and Flow Cytometry Analysis

After being subject to both low (controls) and HFD diets for 8 weeks, mice were terminated and 200 µL of venous blood was collected using the tail bleeding method. Venous blood was collected into 3.2% citrate coated microtainer tubes (Sigma Aldrich, St Louis, Missouri, USA). Moreover, the Beckman Coulter Ac T diff^TM^ analyser (Beckman Coulter, Brea, CA, USA.) was used to measure the baseline (before administration of experimental diets) haematological parameters as per the manufacturer’s protocol.

### 2.3. Instrument Set-Up and Optimization

The BD FACSCanto II flow cytometer (BD Bioscience, Franklin lakes, NJ, USA) was used, and the cytometer set-up and tracking (CST) beads (BD Bioscience, Franklin lakes NJ, USA) were used to perform internal quality control (QC) as per manufacturer’s protocol. To compute and compensate for spectral overlap, BD ™ Compbead compensation particles (BD Bioscience, Franklin lakes NJ, USA) were used. In addition, SPHERO ™ 6-peak Rainbow calibration particles (BD Bioscience, Franklin lakes NJ, USA) were used daily as QC for the median fluorescence intensity (MFI).

### 2.4. Measurement of Baseline PMA Levels

The measurement of baseline PMA levels was performed within 30 min after blood collection. Briefly, 25 µL of the blood was stained with 2.5 µL (ratio 1:10) of the anti-mouse monoclonal antibody cocktail containing CD14-PE (clone: rmC5-3) (monocyte marker), CD41-FITC (clone: MWReg30) (platelet marker) and CD45-BV510 (clone: 30-F11) (leukocyte marker) for 10 min in the dark at room temperature. These samples were fixed using 25 µL of thrombofix (Beckman Coulter, Brea, CA, USA) prior to red blood cell (RBC) lysis. The samples were then lysed with 350 µl FACSLyse lysis buffer (BD Bioscience, NJ, USA) for 15 min in the dark at room temperature. This was then analysed on the BD FACSCanto II flow cytometer.

### 2.5. Measurement of PMA Post-Stimulation with ADP

To investigate the role of agonist-activated platelets in the formation of PMAs under HFD feeding conditions, ADP (adenosine diphosphate) was used to stimulate platelets, whilst PMA levels were determined by flow cytometry. Briefly, 25 µL of the citrated blood was incubated with 10 µL (20 µM) ADP for 15 min and then fixed with 25µl of thrombofix. The sample was then stained with 2.5 µL (ratio 1:10) of anti-mouse monoclonal antibody cocktail containing CD14-PE PE (clone: rmC5-3), CD41-FITC (clone: MWReg30) and CD45-BV510 (clone: 30-F11) (BD Bioscience, NJ, USA) and incubated for 10 min at room temperature in the dark. The analysis was then done on the BD FACSCanto II flow cytometer.

### 2.6. The Gating Strategy for the Enumeration of PMAs

**Figure 1:**
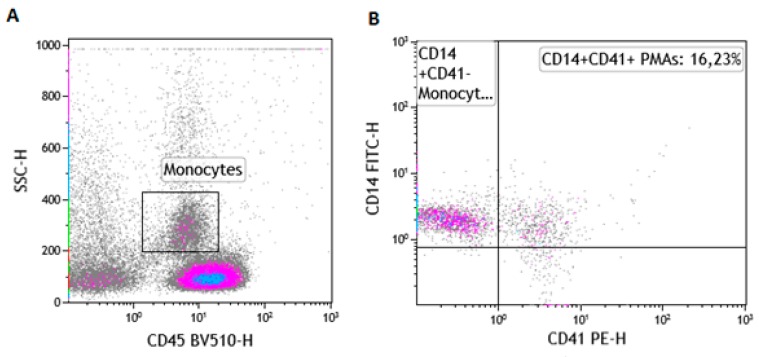
Gating strategy for the enumeration of platelet monocyte aggregates (PMAs). (**A**) illustrates the use of the pan leucocyte marker (CD45) and the side scatter (SSC) properties to identify the monocyte population in whole blood using a control sample. (**B**) illustrates the enumeration of platelet monocyte aggregates (CD45+CD14+CD41+).

The pan-leukocyte marker (CD45) was used to identify leukocyte populations. Amonocyte-specific marker (CD14) was used to identify monocytes. In addition, CD41 was used to identify platelet-bound monocytes and enumerate PMAs (Figure 1A,B).

### 2.7. Statistical Analysis

Statistical analysis was performed using GraphPad Prism 5 (GraphPad Software, San Diego, California, USA). Non-parametric and parametric data were analysed using the Mann-Whitney test and unpaired *t*-test respectively. Non-parametric data was reported as the median IQR. Parametric data was reported as the mean ± standard deviation (SD). A *p* < 0.05 was considered as statistically significant.

## 3. Results

### 3.1. The Impact of HFD on Baseline Metabolic Parameters and Glucose Tolerance

Although there were no significant differences between the body weights of the HFD group when compared to the LFD group, mice kept on the HFD for 8 weeks displayed impaired glucose tolerance and markedly increased insulin levels when compared to animals in the control group (Table 2). Furthermore, baseline haematological markers showed varied modulation between the HFD group and the controls (Table 2). In particular, haematological markers such as RBC count (*p* = 0.0178), haematocrit (*p* = 0.0433) and mean cell volume (*p* = 0.0025) showed a significant difference when the HFD group was compared to the controls (Table 2).

### 3.2. The Impact of HFD on Platelet-Monocyte Aggregates

**Figure 2:**
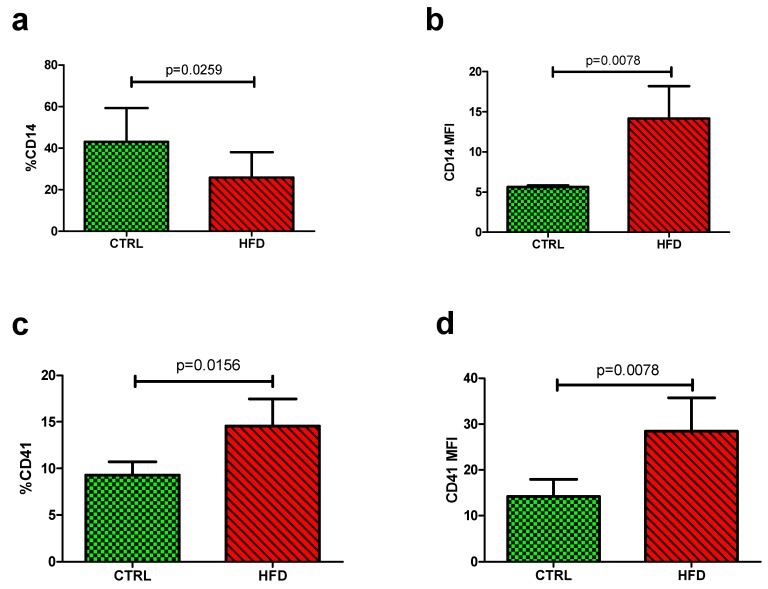
Baseline monocyte and platelet-monocyte aggregate levels between the control (CTRL) group and the high-fat diet (HFD) group. (**a**) Monocyte levels (%CD14) were significantly lower in the HFD group compared to the control group at baseline, *p* = 0.0259. (**b**) The qualitative measurement (CD14 MFI) however was increased in the HFD compared to the control group, p=0.0078. PMAs were determined by the level of platelet-bound monocytes. (**c**) The HFD group had higher levels of PMA compared to the control group at baseline measurement, *p* = 0.0156. (**d**) Similarly, the qualitative measurement (CD41 MFI) was increased in the HFD compared to the control group, p=0.0078. PMA: platelet-monocyte aggregate; HFD: high-fat diet; MFI: median fluorescence intensity.

The levels of monocytes were determined by measuring the levels of CD14 expression from each sample. The HFD (25.93 ± 12.17) showed lower quantitative levels of monocyte (%CD14) compared to the control group (42.98 ± 16.34, *p* = 0.0259) (Figure 2a). In contrast, the qualitative median fluorescence intensity (MFI) was elevated in the HFD group (14.18 ± 18.80) compared to the control group (5.66 ± 0.51, *p* = 0.0078) (Table 3, Figure 2b).

While other parameters did not show significant changes, baseline levels of platelet-monocyte aggregates (%CD41) were markedly increased in the HFD group (14.55 ± 13.66) compared to the control group (9.28 ± 4.05, *p* = 0.0156) (Figure 2c). The qualitative analysis measured using the median fluorescence intensity (MFI) also showed increased levels in the HFD group (28.45 ± 34.13) compared to the control group (14.19 ± 10.64, *p* = 0.0078) (Table 3, Figure 2d).

The measurement of PMA levels was performed within 30 min after blood collection. Briefly, blood was stained with the anti-mouse monoclonal antibody cocktail containing CD14-PE (monocyte marker) and CD41-FITC (platelet marker) for 10 min in the dark at room temperature. The samples were then lysed with FACSLyse lysis buffer before being analysed on the BD FACSCanto II flow cytometer.

### 3.3. The Impact of HFD on the Modulation of PMAs Post-Stimulation with ADP

Results from this study showed that post-stimulation with ADP induced a significant decrease in %CD14 in the control group 17.99 (8.46–20.31), when compared to the unstimulated levels 63.16 (61.10–63.80), *p* = 0.0074 (Table 3). There were no significant differences in post-stimulation with ADP in qualitative measurements (CD14 MFI) (Table 3). Moreover, PMA levels (%CD41) were significantly increased in the control group post-stimulation with ADP 25.97 (20.02–33.22), when compared to unstimulated levels 12.55 (12.49–16.34), *p* = 0.0438 (Table 3).

Furthermore, it was also clear that the HFD group showed a significant decrease in the %CD14 post-stimulation with ADP 14.37 (8.430-18.98) compared to unstimulated levels 13.52 (4.590–16.08), *p* = 0.0405. Interestingly there was no significant differences between the unstimulated PMA levels 29.96 ± 11.40 and post-ADP stimulation 28.94 ± 10.79, *p* = 0.4375 (Table 3).

## 4. Discussion

The aim of this study was to evaluate the impact of HFD feeding in PMA formation using a mouse model. Importantly, the C57BL/6 mice used were ideal for this study since they have already been shown to develop glucose intolerance when fed on a HFD [14]. This study also aimed at elucidating a link between inflammation and the hypercoagulable state observed under conditions of metabolic dysregulation. Overall, this study was able to demonstrate that under HFD conditions, activated platelets readily interact with monocytes, forming PMAs, which have been described as early markers for atherosclerosis in T2DM [15]. The formation of these aggregates with short-term HFD feeding may indicate the hyperreactive nature of platelets which characterize the early onset of a T2DM condition. We could demonstrate that activated platelets were capable of forming these interactions by flow cytometry measurements of platelet bound monocytes. This is in agreement with previous studies which demonstrated that activated platelets interact with monocytes via P-selectin and its counter-receptor PSGL-1 expressed on the surface of monocytes [3,4,16]. Interestingly, in addition, it was clear that the haematological indices measured, which included the RBC and platelet count as well as the plateletcrit, were reduced in HFD-fed mice, when compared to the controls. Consistent with previous evidence, anaemia is common in overweight and obese states [17,18,19,20], however, little is known on the relationship with thrombocytopenia. Thus, this suggests that additional studies are necessary that aim to understand the relationship between reduced haematological parameters in obesity, especially its impact in the development of cardio-metabolic complications. On the other hand, ultrastructural changes in the morphology RBCs have been associated with thrombotic complications in patients living with T2DM [21]. Notably, HFD promotes the activation of pro-inflammatory monocytes and RBC dysfunction [22]. This results in increased membrane phosphatidylserine exposure, which promotes apoptosis and increased phagocytosis of RBCs by activated monocytes [23,24]. Taken together, the HFD-induced alterations in the RBC indices may be due to an increased clearance of RBC by activated monocytes and could be driven by chronic inflammation.

Nonetheless, to the best of our knowledge, our study is the first to assess the impact of HFD feeding of PMA formation using an animal model, which could significantly enhance our current understanding on the susceptibility of the vasculature to abnormally enhanced platelet activation.

The animal experimental model showed that HFD-fed mice exhibited elevated levels of PMAs, indicating increased interactions between platelets and monocytes when compared to the controls. The qualitative increase of the PMAs also reiterates the increased levels of platelet-monocyte interactions with HFD feeding which may promote thrombosis. This is indeed supported by a previous study showing increased PMA levels in individuals who already had coronary artery disease [16]. Nevertheless, individuals with myocardial infarctions already exhibit high levels of PMAs [25,26]. It has been suggested that increased levels of PMA as well as platelet-neutrophil aggregates under dysregulated metabolic conditions such as T2DM further highlight the importance of the platelet-monocyte interactions in the progression of the pro-thrombotic state [12].

In response to ADP stimulation, HFD feeding promoted the formation of PMAs indicating the hyperreactive nature of platelets in this disease state. ADP is a platelet agonist which activates the P2Y12 pathway resulting in its translocation of P-selectin from the alpha granules to the cell surface [25,27,28]. It is already acknowledged that activated platelets form interactions with monocytes, and this process is mediated by the binding of P-selectin to its receptor PSGL-1 on the surface of these cells [3]. Subsequently, platelet-bound monocytes are activated and differentiated into pro-inflammatory monocytes. This is accompanied by an increase in the expression of CD11b, a marker of monocyte activation [3,21,29]. To that effect, our study showed a decrease in CD14 monocyte marker, indicating a shift in the monocyte phenotype to the pro-inflammatory form CD14++ which can be explained by the higher qualitative analysis (CD14 MFI). Monocytes exhibit distinct heterogeneous features which can be classified using flow cytometry into classic (CD14++CD16-), intermediate (CD14++CD16+) and non-classic (CD14+CD16++). The intermediate monocyte subtype expresses genes associated with inflammation and angiogenesis [30].

Overall, the hyperreactive nature of platelets with short-term exposure to HFD feeding in mice may be associated with increased PMA formation, the early marker of atherosclerosis that is known to promote a pro-inflammatory state [14,31]. A drawback of this study may be that the levels of pro-inflammatory markers produced as a result of PMA formation were not determined. Further, studies evaluating the impact of HFD on pro-inflammatory response as a result of platelet-binding interactions will give a better understanding of implicated mechanisms of action, especially providing an insight into its influence in the transmigration of monocytes into metabolic tissue. In any case, studies looking at the direct effect of HFD on markers of atherosclerosis and inflammation under varied metabolic conditions are also necessary.

## Figures and Tables

**Table 1:** An overview of diet composition (g/kg) for both control and high-fat diet-fed mice.

Ingredients	Low-fat Diet (Control) ^a^	High-fat Diet ^b^
**Casein, 30 mesh**	200.00	200.00
**L-Cystine**	3.00	3.00
**Corn starch**	506.20	-
**Lodex 10**	125.00	125.00
**Sucrose**	72.80	72.80
**Solka Floc, FCC200 (Fiber)**	50.00	50.00
**Soybean Oil**	25.00	25.00
**Lard**	20.00	245.00
**Mineral mix S10026B**	50.00	50.00
**Choline Bitartrate**	2.00	2.00
**Vitamin mix V10001C**	1.00	1.00
**Dye, Yellow FD&C #5, Alum. Lake 35–42%**	0.04	-
**Dye, Blue FD&C #1, Alum. Lake 35–42%**	0.01	0.05

^a^ The low-fat diet obtained from Research Diets Inc (#D12450J, rodent diet with 10% kcal% fat) provided 3.82 kcal/g from 20%, 70%, and 10% of protein, carbohydrate, and fat, respectively. ^b^ The high-fat diet (HFD) obtained from Research Diets Inc (#D12492, rodent diet with 60% kcal% fat) provided 5.21 kcal/g from 26.2%, 26.3%, and 34.9% of protein, carbohydrate, and fat, respectively. Typical analysis of cholesterol in lard = 0.72 g/kg.

**Table 2:** An overview of metabolic and haematological parameters between high-fat diet-fed mice and controls.

Parameter	Control (*n* = 8)	High-fat Diet (*n* = 8)	*p*-Value
**Weight gain (g)**	25.0 ± 2.5	26.0 ± 1.9	0.43
**Glucose levels (mmol/L)**	6.1 (5.4–6.9)	8.7 (8.5–9.2)	**0.008**
**AUC mmol/L × 120 min**	636.0 (559.9–702.0)	765.0 (715.5–784.5)	**0.032**
**Insulin levels (µIU/mL)**	4.5 (4.4–4.6)	4.8 (4.6–8.1)	**0.026**
**White cell count (10^3^/µL)**	5.35 (3.68–9.00)	7.50 (4.80–8.40)	0.4699
**Red blood cell count (10^6^/µL)**	7.17 (7.04–7.69)	6.910 (5.53–7.17)	**0.0178**
**Haemoglobin (g/dL)**	25.85 (20.00–29.23)	22.40 (16.75–25.00)	0.6683
**Haematocrit (%)**	31.10 (30.05–33.30)	29.00 (23.00–31.40)	**0.0433**
**Mean cell volume (fL)**	43.00 (43.00–43.75)	42.00 (41.00–44.00)	**0.0025**
**Platelet count (10^3^/µL)**	782.9 ± 206.4	697.2 ± 151.1	0.5789
**Mean platelet volume (fL)**	5.30 (5.03–5.50)	5.20 (5.10–5.40)	0.6957
**Neutrophil count (%)**	7.75 (7.00–9.48)	8.000 (6.90–9.30)	0.9640
**Lymphocyte count (%)**	89.85 (88.15–90.73)	89.20 (87.80–90.50)	0.5271
**Monocyte count (%)**	1.96 ± 0.24	2.05 ± 0.56	0.6840
**Basophil (%)**	0.25 (0.13–0.50)	0.2000 (0.10–0.80)	0.7997
**%CD14**	31.05 (30.74–31.61)	31.13 (29.65–35.78)	0.4695
**CD14 MFI**	5.325 (5.14–5.45)	5.59 (5.32–5.79)	**0.0350**
**%CD41**	7.06 (5.81–8.06)	5.82 (4.43–6.83)	0.2757
**CD41 MFI**	6.36 (6.04–7.52)	6.12 (5.81–6.74)	0.7892

Data presented as mean ± SD and median (IQR); *p* < 0.05 shown in boldface; MFI: Median fluorescence intensity.

**Table 3:** Platelet monocyte aggregate (PMA) formation following stimulation with 20 µM of adenosine diphosphate.

**Control diet**	**Unstimulated (*n* = 3)**	**Post-ADP (*n* = 3)**	***p*-value**
**%CD14**	63.16 (61.10–63.80)	17.99 (8.46–20.31)	**0.0074**
**CD14 MFI**	6.13 (5.98–6.55)	18.00 (12.53–64.61)	0.2596
**%CD41**	12.55 (12.49–16.34)	25.97 (20.02–33.22)	**0.0438**
**CD41 MFI**	25.84 (22.45–31.69)	34.22 (24.06–41.80)	0.2854
**High-fat diet**	**Unstimulated (*n* = 8)**	**Post-ADP (*n* = 7)**	***p*-value**
**%CD14**	13.52 (4.590–16.08)	14.37 (8.430–18.98)	**0.0405**
**CD14 MFI**	16.53 (11.28–45.47)	20.32 (14.66–73.78)	0.3125
**%CD41**	29.96 ± 11.40	28.94 ± 10.79	0.4375
**CD41 MFI**	67.22 ± 28.19	40.08 ± 14.95	0.0938

*p* < 0.05 shown in boldface.
